# External validation of risk scores and multivariate models for the diagnosis of community-acquired pneumonia in outpatients

**DOI:** 10.1080/13814788.2026.2628370

**Published:** 2026-02-20

**Authors:** Mark Ebell, Dan J. Merenstein, Bruce Barrett, Theo Verheij, Paul Little

**Affiliations:** a Department of Family Medicine, Michigan State University, East Lansing, MI, USA; b Department of Family Medicine, Georgetown University, Washington, DC, USA; c Department of Family Medicine and Community Health, University of Wisconsin, Madison, WI, USA; d Julius Centre for Health Sciences and Primary Care, University Medical Center Utrecht, Utrecht, Netherlands; e Primary Care and Population Sciences, University of Southampton, Southampton, UK

**Keywords:** Community-acquired pneumonia, lower respiratory tract infection, risk score, clinical prediction rule, primary care

## Abstract

**Background:**

While several risk scores for the diagnosis of community-acquired pneumonia (CAP) have been developed, they require prospective external validation.

**Objectives:**

To externally validate existing prediction models, risk scores, and heuristics for the diagnosis of CAP in adults.

**Methods:**

The Enhancing Antibiotic Stewardship in Primary Care (EAST-PC) study recorded signs, symptoms, demographics, and vitals in 718 adults presenting to primary or urgent care clinics with acute lower respiratory tract infection between 2019 and 2023. C-reactive protein (CRP) was available for 575. The diagnosis of CAP was based on the clinician diagnosis and/or chest radiograph. Literature was searched for previous risk scores. Using the EAST-PC population, the area under the receiver operating characteristic curve (AUROCC), calibration curves, and percentage with CAP in each risk group were calculated for each risk score.

**Results:**

We identified 11 studies describing 4 risk scores, 9 multivariate models, and 5 simple heuristics. The Genomics to Combat Resistance Against Antibiotics in Community-acquired LRTI in Europe (GRACE) risk score using the absence of a runny nose, the presence of breathlessness, crackles, diminished vesicular breathing, heart rate > 100/min, temperature >37.8 °C, and CRP > 30 mg/L was the most accurate (AUROCC 0.81). It classified 280 patients as low (0.7% CAP), 265 as moderate (5.7%) and 30 as high risk (33.3%) for CAP. The GRACE score without CRP performed similarly. Other risk scores had poor calibration or failed to accurately classify patients as low or high risk.

**Conclusions:**

The previously derived GRACE risk scores were successfully externally validated in a contemporary US outpatient population.

## KEY MESSAGES

- Two risk scores using clinical exam findings (with and without CRP) had acceptable accuracy for the diagnosis of community-acquired pneumonia (CAP).
- Their use classifies about half of patients with lower respiratory tract infections as very low risk for CAP
- Further validation studies would be desirable in primary care settings.

## Introduction

Previous studies have found that approximately 3% to 5% of adults presenting to a primary care physician with a lower respiratory tract infection (LRTI) have radiographically confirmed community-acquired pneumonia (CAP). This is based on studies where all patients underwent chest radiography (CXR) [1–4]. However, chest radiographs are not always available on site or may be a burden for patients to obtain in terms of convenience or cost. Guidelines from the British Thoracic Society, the UK National Institute for Clinical Excellence and the Infectious Disease Society of America acknowledge that radiography is often impractical in primary care settings, where the diagnosis is often made clinically, but recommend it for patients with a high level of suspicion and when the diagnosis may be in doubt [5–7]. Thus, the ability to reliably identify patients at lower or higher risk of an abnormal chest radiograph (CXR) could be useful in the outpatient setting.

Several groups of researchers have proposed risk scores to stratify primary care outpatients for their likelihood of CAP using signs, symptoms, and simple blood tests like C-reactive protein (CRP) [1–4,8–11]. Two previous studies that obtained a chest radiograph in all participants have attempted to validate these published risk scores. The first evaluated 6 risk scores in a population of 145 Dutch outpatients with abnormal lung exam and findings suggestive of pneumonia, of whom 26 had radiographic CAP [12]. The second validation study used data from 2,820 outpatients in 11 European countries presenting with acute LRTI in primary care as part of the Genomics to combat Resistance against Antibiotics in Community-acquired LRTI in Europe project (GRACE). In the GRACE population, 5% had radiographic pneumonia, and they used their data to attempt to validate 6 previous risk scores [4]. In both studies, the best performing risk score was developed by Hopstaken and colleagues [10] and included signs, symptoms, and CRP. It had an area under the receiver operating characteristic curve (AUROCC) between 0.69 and 0.71. The GRACE investigators also proposed two novel risk scores for the diagnosis of CAP, one based on six signs and symptoms and one adding CRP.

The Enhancing Antibiotic Stewardship in Primary Care (EAST-PC) study identified 718 primary and urgent care patients at 3 sites in the United States who presented with acute cough and one or more symptoms of LRTI [13–15]. An explicit aim of the study was to gather predictors needed to validate previously published risk scores for the diagnosis of CAP. In this paper, we report the results of our attempt to validate previously published risk scores for diagnosis of CAP in a contemporary US outpatient population.

## Methods

This was a prospective external validation of previously published risk scores, models and heuristics for the diagnosis of CAP in outpatient adults. We followed TRIPOD recommendations for validation studies and a checklist is provided in Supplementary Appendix A [16].

### Sponsorship and funding

EAST-PC was a prospective observational study of adult outpatients with acute cough. The study was sponsored by the U.S. Agency for Healthcare Research and Quality (#1R01HS025584-01A1). All participants signed an informed consent form, and the study was approved by the Western Institutional Review Board (IRB, #1253415) and the IRBs of each participating institution. The design of the EAST-PC study has been described in detail previously and is summarised below [13,14].

### Identification of previous diagnostic risk scores for CAP

We first compiled risk scores for CAP in outpatients with RTI that had been identified by the authors for validation in the EAST-PC dataset as part of their original grant application. We also looked for additional studies by reviewing previous external validation studies and performing a search of PubMed for risk scores and models to diagnose CAP in outpatients, using a published search strategy for primary care clinical prediction rules (Supplementary Appendix B) [17],

Inclusion criteria included studies that reported a heuristic, risk score, or multivariate model to diagnose CAP in the outpatient or emergency department (ED) setting. The model or risk score had to use only predictor variables available in the EAST-PC dataset. We excluded studies in specialised populations (e.g. patients with HIV disease, post-operative, cancer, stroke, or post-transplant), studies of pneumonia in hospitalised patients, studies predicting prognosis, severity or mortality, and risk scores for specific pathogens such as *Legionella pneumonia* or *Mycoplasma pneumoniae*.

The PubMed search was reviewed by the lead author (MHE) to identify potentially relevant studies to review as full text. These full-text studies were reviewed in parallel by two investigators to identify any additional studies meeting the inclusion criteria. Studies performing external validation or existing models were also identified. The risk scores or multivariate models and their diagnostic accuracy in the original study as well as in any validation studies was abstracted by the lead author and reviewed by at least one of the co-authors for accuracy.

### Participants in the external validation (EAST-PC) study population

Adults aged 18 to 75 years presenting to primary and urgent care clinics in Athens, Georgia, Washington, D.C., and Madison, Wisconsin, with acute cough were included. At enrolment, all patients reported a cough for no more than 14 days and at least one of: measured fever or feverishness, shortness of breath, myalgias, sputum, chest pain, chest congestion, chills, or sweats. Patients who had taken an antiviral, antibiotic, or corticosteroid in the previous 28 days, those with serious immunodeficiency, those who were receiving cancer chemotherapy, or taking systemic steroids or other immunosuppressive drugs were excluded. Patients with mild to moderate asthma could be included, but those with more severe chronic lung disease including COPD were excluded. Data were collected between June 2019 and April 2023.

### Baseline data collection

Demographic information, symptoms, and comorbidities were collected at baseline. Symptoms were rated by the patient as absent, mild, moderate or severe. Unless otherwise specified by a risk score, a symptom was considered to be present if the patient reported at least moderate severity. All symptoms were patient-reported to study research assistants. Clinical signs were evaluated by the patient’s clinician and recorded on a separate data collection card. Vital signs were obtained from the electronic health record, and CRP was obtained for study use only using the QuikRead Go device (Orion Diagnostica, Finland).

### Criteria for diagnosis of community-acquired pneumonia

The EAST-PC protocol did not include a chest radiograph for all participants. We therefore used a pragmatic approach, accepting either a clinical diagnosis of CAP by the treating physician or abnormal chest radiograph or both to determine whether a patient had been diagnosed with CAP. Radiologists were blinded to clinical data. This is consistent with guidelines acknowledging that it is inappropriate to obtain a CXR in all patients with LRTI or clinically suspected CAP [5–7]. Ultimately, 29 of 718 patients were diagnosed with CAP (4.0%), which is similar to the prevalence reported by other primary care studies [1,4].

### Analysis

Some studies reported only a logistic model, while some also simplified the model into a point-based risk score or a simple heuristic. Where reported by the original study, the proportion of patients with pneumonia in risk groups (e.g. low, moderate, high or by the number of predictors) were calculated using the cut-offs proposed in the original derivation studies. The prespecified goal was to identify risk groups falling below a previously reported test threshold of 10% and above the treatment threshold of 40% [18,19].

Consistent with recommendations from experts in the field of modelling, we report both discrimination and calibration in addition to diagnostic accuracy [20,21]. Discrimination was assessed where possible by calculating the area under the receiver operating characteristic curve (AUROCC, also known as the c-statistic) using the full regression model or risk score. Calibration was determined by drawing calibration curves using the pmcalplot package. All analyses were performed using Stata version 18.5 (StataCorp, College Station, Texas).

## Results

### Patient characteristics in the EAST-PC external validation population

A total of 718 patients were recruited and had baseline data for patient-reported symptoms, physician-assessed signs, and vital signs. These data were recorded for all participants at baseline with no missing data. A valid CRP result was available for 575 patients (105 patients had an invalid result by the device, and 38 patients were recruited during a period when CRP supplies were not available due to pandemic disruptions).

Characteristics of EAST-PC study participants are summarised in Table 1. The mean age was 39 years, with a range of 18 to 75 years. Patients had a cough for a median of 4 days prior to their index visit. The most common symptoms rated moderate to severe by participants were cough (75.1%), feeling generally unwell (72.1%), fatigue (67.5%), and coryza (54.0%). A total of 29 patients (4.1%) had clinically or radiographically diagnosed pneumonia.

**Table 1.** Characteristics of 718 participants in the enhancing antibiotic stewardship in primary care (EAST-P study of outpatients with lower respiratory tract infection.

Patient characteristic	Number (%)
Patient age (mean, range)	38.9 years, 18 to 74
Female sex	469 (65.3%)
Duration of cough prior to presentation in days (median, interquartile range)	4 (3 to 7)
Clinically or radiographically diagnosed pneumonia	29 (4.1%)
Symptoms rated moderate or severe at baseline by at least 30% of participants	
Cough	539 (75.1%)
Feeling generally unwell	518 (72.1%)
Fatigue	485 (67.5%)
Coryza	388 (54.0%)
Headache	290 (40.4%)
Myalgia	265 (36.9%)
Chest congestion	262 (36.5%)
Chest pain or ache with cough	254 (35.4%)
Presence of specific symptoms	
Cough causing shortness of breath or light-headedness	358 (49.9%)
Felt warm or feverish on every day of illness	334 (46.5%)
Cough causing nausea or vomiting	312 (43.5%)
Double-sickening	286 (39.8%)
Measured fever ≥ 101 F (37.8 C) at home	88 (12.3%)

### Identification of published risk scores for diagnosis of CAP in outpatients

Nine studies were known to the authors from their previous work. A review of previous external validation studies [4,12,22,23] identified one additional risk score but it required a white blood cell count, which was not available in our dataset [24]. Finally, the PubMed search identified 364 abstracts, of which 9 were reviewed in full text and 2 met the inclusion criteria [23,25]. In all, 11 studies describing 4 risk scores, 9 multivariate models, and 5 simple heuristics for outpatient diagnosis of CAP met our inclusion criteria. Characteristics of the included studies are summarised in Table 2, as well as the risk scores, heuristics, and models themselves.

**Table 2.** Previously published risk scores and models for predicting the likelihood of pneumonia in outpatient adults.

Author, Year	Population and setting	Pneumonia/ Total (#)	Approach	Risk score or multivariate equation
Diehr, 1984 [1]	All adults with acute cough in emergency department (ED); all received chest x-ray (CXR)	48/1819 (2.6%)	Risk score	Rhinorrhea (−2), sore throat (−1), night sweats (+1), myalgia (+1), sputum (+1), respiratory rate >25/minute (+2), temperature >37.7 °C (+2)
Gennis, 1989 [26]	Adults with CXR ordered in ED for suspected community-acquired pneumonia (CAP)	118/308 (38%)	Simple heuristic	Low risk for CAP if temperature ≤ 37.8 C, pulse ≤ 100/minute and respiratory rate ≤ 20/minute.
Groeneveld, 2019 [11]	Adults with CXR ordered from primary care for suspected CAP	30/249 (12%)	Multivariate models	Model 1: *p* = 1 / (1 + e^-Y^) where Y = −4.492 + (1.142 x absence of runny nose) + (2.55 x feel ill)
				Model 2: *p* = 1 / (1 + e^-Y^) where Y = −4.797 + (1.230 x absence of runny nose) + (2.378 x feel ill) + (1.572 x CRP > 30 mg/L)
Heckerling, 1990 [2]	Adults with CXR ordered in ED for acute cough or fever	135/914 (15%)	Risk score	1 point each for: absence of asthma, temperature > 37.8 C, heart rate > 100/minute, decreased breath sounds, rales
			Multivariate model	*p* = 1 / (1 + e^-Y^) where Y = −1.705 + (0.494 x temperate > 37.8 C + (0.428 x heart rate > 100/minute) + (0.638 x decreased breath sounds) + (0.658 x rales) + (0.692 x absence of asthma)
Hopstaken, 2003 [10]	Adults with acute cough and other features of lower respiratory tract infection (RTI); all received CXR	32/246 (13%)	Multivariate models	Model 1 (signs and symptoms): *p* = 1 / (1 + e^-Y^) where Y = −2.74 + (1.02 x dry cough) + (1.78 x diarrhoea) + (1.13 x temperature ≥ 38 C)
				Model 2 (adding c-reactive protein [CRP]): *p* = 1 / (1 + e^-Y^) where Y = −4.15 + (0.91 x dry cough) + (1.01 x diarrhoea) + (0.64 x temperature ≥ 38 C) + (2.87 x CRP > 20 mg/L)
			Simple heuristic	Low risk for CAP if no more than one of diarrhoea, dry cough, or temperature ≥ 38 C AND CRP < 20 mg/L
Melbye, 1992 [3]	Adults in ED with RTI; 181/402 received CXR	20/402 (5%)	Multivariate model	*p* = 1 / (1 + e^-Y^) where Y = (4.7 x subjective fever and duration ≥ 1 week) + (5.0 x dyspnoea severe) + (8.2 x chest pain severe) + (0.9 x crackles) – (4.5 x coryza) – (2.1 x sore throat)
O’Brien, 2006 [25]	Adult outpatients with acute RTI and with (*n* = 350) or without (*n* = 350) CAP by CXR (case-control design)	350/700 (50%)	Simple heuristic	Low risk for CAP if none of abnormal lung exam, temperature ≥ 38.0 C, heart rate ≥ 100/minutes or respiratory > 20 breaths/minute
Singal, 1989	Adults in ED with CXR ordered for suspicion of pneumonia	40/255 (16%)	Multivariate model	*p* = 1 / (1 + e^-Y^) where Y = −3.095 + (1.214 x cough) + (1.007 x fever) + (0.823 x crackles)
			Simple heuristic	Low risk for CAP if no fever, cough, or crackles
Steurer, 2011 [9]	Adults in primary care with acute cough and fever; all received CXR	127/621 (20%)	Simple heuristic *	Low risk if CRP ≤ 10 mg/L OR CRP 11 to 50 mg/L and no dyspnoea or daily fever since onset of cough
Tse, 2019 [23]	Adults in ED with fever and respiratory symptoms who had a CXR ordered	100/537 (19%)	Risk score	Temperature ≥ 40 C (+2), fever > 3 days (+2), abnormal breath sounds (+1), oxygen saturation ≤ 96% (+1), age ≥ 65 (+1), history of pneumonia (+1), sore throat (−2)
Van Vugt, 2013 [4]	Adults in primary care with acute cough; all received CXR	140/2820 (5%)	Risk score	1 point each for: absence of runny nose, presence of breathlessness, crackles, diminished vesicular breathing, heart rate > 100/min, temperature >37.8 °C, and CRP > 30 mg/L
			Multivariate models	Model 1 (signs and symptoms): *p* = 1 / (1 + e^-Y^) where Y = −3.984 + (0.446 × breathlessness) + (0.698 × absence of runny nose) + (0.596 × diminished vesicular breathing) + (1.404 × crackles) + (0.961 × tachycardia) + (0.980 × temperature >37.8 °C)
				Model 2 (adding CRP): *p* = 1 / (1 + e^-Y^) where Y = −3.984 + (0.446 × breathlessness) + (0.698 × absence of runny nose) + (0.596 × diminished vesicular breathing) + (1.404 × crackles) + (0.961 × tachycardia) + (0.980 × temperature >37.8 °C) + (0.130 x CRP/10)

* Heuristic for Steurer, 2011 was based on a simple classification and regression tree (CART) derived algorithm.

### Validation of previously published risk scores

Four studies were identified that performed external validation of published risk scores [4,12,22,23]. An additional external validation study was identified but it did not report AUROCC [27]. The AUROCC for the external validations in these studies and in the EAST-PC validation population are summarised in Table 3. The most widely validated risk score was that of Heckerling with the AUROCC ranging from 0.57 to 0.88. Hopstaken’s multivariate model that included CRP had AUROCC’s of 0.69 and 0.71 in two external validation studies. The AUROCC was generally consistent between the GRACE and EAST-PC studies, 0.70 vs 0.71 for the signs and symptoms score and 0.78 vs 0.81 for the risk score, adding CRP. The GRACE risk scores with and without CRP by van Vugt and colleagues had the highest AUROCC and it was similar between the original derivation and EAST-PC populations.

**Table 3.** Area under the receiver operating characteristic curve (AUROCC) and 95% confidence intervals for included derivation and validation studies.

		Previous external validation studies				EAST-PC validation
Author, Year (risk score or model)	Derivation study	**Flanders, 2004 [22**]	**Graffelman, 2007 [12**]	**Tse, 2019 [23**]	**Van Vugt, 2013 [4**]	
Diehr, 1984 (risk score) [1]	NR			0.72 (0.68–0.76)	0.67 (0.62–0.72)	0.57 (0.46–0.67)
Groeneveld, 2019 (model 1 signs and symptoms) [11]	NR					0.62 (0.51–0.73)
Groeneveld, 2019 (model 2 adding c-reactive protein [CRP])	NR					0.64 (0.52–0.76)
Heckerling, 1990 (model) [2]	0.82 (0.78–0.86)	0.88 (NR)	0.63 (0.50–0.75)	0.57 (0.52–0.61)	0.65 (0.59–0.70)	0.64 (0.52–0.76)
Hopstaken, 2003 (model 1 signs and symptoms) [10]	0.70 (NR)		0.62 (0.50–0.75)		0.55 (0.50–0.61)	0.56 (0.42–0.69)
Hopstaken, 2003 (model 2 adding CRP)	0.80 (NR)		0.69 (0.58–0.80)		0.71 (0.66–0.76)	0.68 (0.44–0.91)
Melbye, 1992 (model) [3]	NR		0.49 (0.37–0.62)		0.65 (0.60–0.70)	0.67 (0.54– 0.80)
Singal, 1989 (model) [8]	NR		0.58 (0.45–0.70)		0.68 (0.62–0.73)	0.79 (0.71–0.87)
Tse, 2019 (risk score) [23]						0.59 (0.49–0.69)
Van Vugt, 2013 (model 1 signs and symptoms) [4]	0.70 (0.65–0.75)					0.71 (0.58–0.84)
Van Vugt, 2013 (model 2 adding CRP)	0.78 (0.74–0.82)					0.81 (0.72–0.89)

NR = not reported; EAST-PC = Enhancing Antibiotic Stewardship in Primary Care study, US data collection from 2019 to 2023.

Table 4 summarises the classification accuracy of the risk scores in the original derivation population and in the EAST-PC population. Risk scores with generally similar classification accuracy in the EAST-PC validation group as in the original derivation study include risk scores from Diehr and colleagues and the GRACE risk score using CRP. The classification accuracy of the GRACE score without CRP (not presented in the original paper by van Vugt) is shown for settings without access to CRP.

**Table 4.** Classification accuracy of risk scores and simple heuristics for the diagnosis of community-acquired pneumonia (CAP) in the original derivation study and in the enhancing antibiotic stewardship in primary care (EAST-PC) external validation populations.

	*n*/total = % CAP in each risk group	
Risk score	Derivation	EAST-PC Validation
Diehr, 1984 (risk score) [1]	Low risk < 1 point: 19/1512 = 1.2% High risk 1+ points: 27/200 = 13.5%	Low < 1 point: 11/533 = 2.1% High 1+ points: 18/185 = 9.7%
Gennis, 1989 (heuristic) [26]	Low risk: 4/40 = 10.0% High risk: 114/268 = 42.5%	Low risk: 20/539 = 3.7% High risk: 9/179 = 5.0%
Groeneveld, 2019 (model 1 signs and symptoms) [11]	Low risk < 2.5%: 0/51 = 0% Moderate risk 2.5%-20%: 16/146 = 11.0% High risk > 20%: 14/45 = 31.1%	Low risk < 2.5%: 2/146 = 1.4% Moderate risk 2.5%-20%: 22/506 = 4.4% High risk > 20%: 5/66 = 7.6%
Groeneveld, 2019 (model 2 adding c-reactive protein [CRP]) [11]	Low risk < 2.5%: 0/49 = 0.0% Moderate risk 2.5%-20%: 8/125 = 6.4% High risk > 20%: 22/68 = 32.3%	Low risk < 2.5%: 2/110 = 1.8% Moderate risk 2.5%-20%: 18/378 = 4.8% High risk > 20%: 7/87 = 8.0%
Heckerling, 1990 (risk score) [2]	0 signs/symptoms: 1/49 = 2.0% 1: 11/327 = 3.4% 2: 28/260 = 10.8% 3: 42/191 = 22.0% 4: 37/67 = 55.2% 5: 15/20 = 75.0%	0 signs/symptoms: 3/108 = 2.8% 1: 14/463 = 3.0% 2: 8/98 = 8.2% 3: 2/46 = 4.4% 4: 2/3 = 67% 5: 0/0
Hopstaken, 2003 (heuristic) [10]*	Low risk: 3/107 = 2.8% High risk: 29/139 = 20.9%	Low risk: 6/79 = 3.6% High risk: 23/639 = 7.6%
O’Brien, 2006 (heuristic) [25]	Low risk: 17/213 = 8.0% High risk: 333/487 = 68.4%	Low risk: 18/434 = 4.1% High risk: 9/141 = 6.4%
Singal, 1989 (heuristic) [8]	Low risk: 3/67 = 4.5% High risk: 37/188 = 19.7%	Low risk: 1/165 = 0.6% High risk: 28/553 = 5.1%
Steurer, 2011 (heuristic) [9]	Low risk by CART**: 0/190 = 0.0% High risk by CART: 127/431 = 29.5%	Low risk by CART: 20/471 = 4.2% High risk by CART: 7/104 = 6.7%
Tse, 2019 (risk score) [23] +	< 0 points: 5/177 = 2.8% 0: 7/106 = 6.6% 1: 10/72 = 13.9% 2: 39/100 = 39.0% 3: 20/51 = 39.2% ≥ 4: 19/31 = 61.3% < 0: 5/177 = 2.8% 0 to 2: 56/278 = 20.1% ≥ 3: 39/82 = 47.6%	< 0 points: 3/151 = 2.0% 0: 8/238 = 3.4% 1: 10/125 = 8.0% 2: 3/110 = 2.7% 3: 4/70 = 5.7% ≥ 4: 1/20 = 5.0% < 0: 3/151 = 2.0% 0 to 2: 21/473 = 4.4% ≥ 3: 5/90 = 5.6%
Van Vugt, 2013 (risk score based on model 2 omitting CRP) [4]	(Results not presented in original study)	0 points: 2/291 = 0.69% 1-2: 18/266 = 6.8% ≥ 3: 7/18 = 38.9%
Van Vugt, 2013 (risk score based on model 2 with CRP) [4]	0 points: 4/572 = 0.7% 1–2: 73/1902 = 3.8% ≥ 3: 63/346 = 18.2%	0 points: 2/280 = 0.71% 1–2: 15/265 = 5.7% ≥ 3: 10/30 = 33.3%

+ Tse, 2019 used “Persistent fever and cough since illness onset” instead of “Fever > 3 days”.

* Hopstaken: Low risk defined as no more than one of diarrhoea, dry cough and temperature≥ 38 C, and CRP < 20 mg/L.

** CART = classification and regression tree.

**Figure 1.**
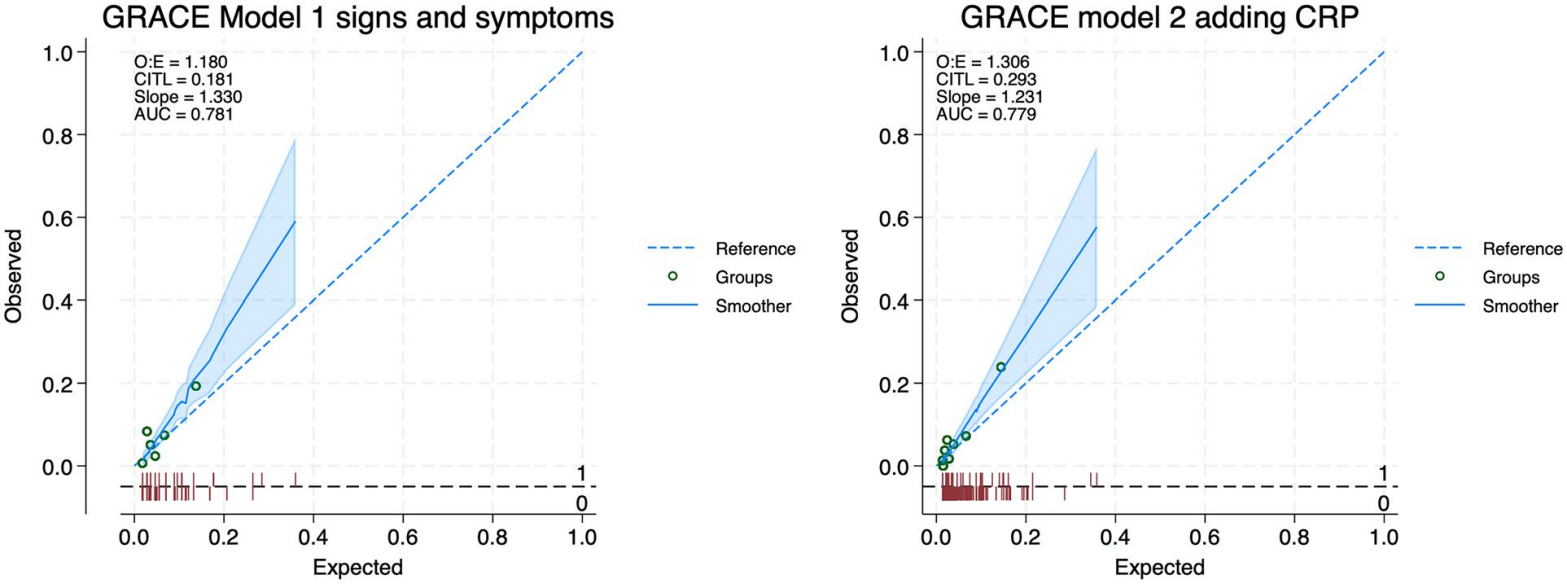
Calibration (observed versus expected) for GRACE model 1 (signs and symptoms) and model 2 (adding c-reactive protein).

Finally, Figure 1a shows the calibration plot for the GRACE Model 1 with signs and symptoms only comparing expected based on GRACE with observed in EAST-PC. Figure 1b shows the calibration plot for the GRACE model 2 with CRP added. Calibration was moderately good for these two models and much better than that observed for any other model or risk score (Supplementary Appendix C).

## Discussion

The risk scores with the best discrimination in the EAST-PC prospective validation population were the GRACE model using signs and symptoms (AUROCC 0.71), the Singal model (AUROCC 0.79), and the GRACE model incorporating CRP (AUROCC 0.81). These models all show good to very good overall accuracy in a contemporary US outpatient population.

A simplified version of the GRACE risk score incorporating CRP gives 1 point each for absence of coryza, crackles on exam, diminished vesicular breathing on exam, pulse > 100/minute, measured fever > 37.8 C, and CRP > 30 mg/L (range 0 to 7 points). Patients in the EAST-PC study population with 0 points (comprising about half of the sample) had a 0.7% risk of CAP, while those with 1 to 2 points a 5.7% risk and those with 3 or more points an 33.3% risk [4]. Thus, GRACE risk score with CRP performed well at identifying of what we judged to be a clinically meaningful number of patients below the test threshold and above the treatment threshold (Table 4).

The Singal risk score had good discrimination as measured by the AUROCC, but it only classified 23% of participants as low risk (0.6% risk of CAP). The Diehr risk score has greater potential utility, classifying three-quarter patients as low risk (2.1% risk of CAP) compared with 9.7% in the high-risk group. It has the advantage of not requiring CRP, which is often not available in the US and some European countries. However, both scores had relatively poor calibration in the EAST-PC population.

Calibration was best for the two GRACE models, although both tended to underestimate the likelihood of CAP at all risk levels in the EAST-PC validation population. Other risk scores and models fared poorly with regard to calibration (Supplementary Appendix C).

Thus, we conclude that the risk score developed using GRACE data by van Vugt and colleagues are the most likely to be useful in clinical practice. While the score with CRP had slightly better discrimination, the classification accuracy of the simplified risk score (Table 4) was similar with and without CRP. While validation in other populations is desirable, it is currently the best choice for helping clinicians assess their patient’s likelihood of CAP and help them target diagnostic testing and treatment.

### Strengths and limitations

Strengths of this study include a diverse, contemporary and geographically varied population in the United States, and prospective data collection of signs, symptoms, vital signs and CRP. The primary limitation of our study is that not all patients underwent a chest radiograph. As noted earlier, though, obtaining imaging in all primary care patients with possible CAP is not recommended by guidelines, and our approach of accepting both clinical and radiographic diagnosis is pragmatic. The fact that our prevalence of pneumonia was similar to that in GRACE (where all patients received a CXR) and the robust discrimination and accuracy of the GRACE risk score in the EAST-PC population further validates this pragmatic approach.

A limitation in some countries is the unavailability of point of care CRP devices in primary care clinics. They are only approved for moderate complexity laboratories in the US, and their use is not reimbursed for general practices in the UK. This is despite the fact that CRP has been shown to be an independent predictor of bacterial rhinosinusitis [28], bacterial lower respiratory infection [15,29], and CAP [4], and that use of CRP can safely reduce inappropriate antibiotic use [30].

### Suggestions for future research

Future studies should be adequately powered, be set in the primary and urgent care setting, gather data at baseline needed to calculate the GRACE and other risk scores, and ideally use imaging for all patients as a diagnostic reference standard. As point of care ultrasound (POCUS) becomes more widely available in the primary care setting, this could provide a less costly, more convenient, and safer alternative to use as the reference standard, with studies showing better accuracy of POCUS than CXR [31] and its use was endorsed by the most recent IDSA guidelines [5].

## Conclusions

Two risk scores developed by van Vugt and colleagues using European GRACE data, have been successfully validated in a contemporary US EAST-PC study population. Additional validation studies are encouraged, as well as development and external validation of accurate risk scores that do not incorporate CRP for settings where it is difficult to access.

## Supplementary Material

Supplemental Material

Supplemental Material

## Data Availability

Data are available upon reasonable request to researchers
