# Supplementary material for: External validation of risk scores and multivariate models for the diagnosis of community-acquired pneumonia in outpatients
Source: Eur J Gen Pract. 2026 Feb 20;32(1):2628370. doi: 10.1080/13814788.2026.2628370 (PMC12927402; doi:10.1080/13814788.2026.2628370)
Supplement: Supplemental Material [file IGEN_A_2628370_SM3948.docx]

**Appendix A. Search strategy to search for outpatient pneumonia risk scores**

("pneumonia"[tiab] OR “infiltrates”[ti]) AND (“prediction of pneumonia”[ti] OR "clinical prediction"[tiab] OR "risk score"[tiab] OR "clinical model*"[tiab] OR "clinical score*"[tiab] OR "decision rule*"[tiab] OR "diagnostic rule*"[tiab] OR "diagnostic score*"[tiab] OR “diagnostic risk score”[tiab] OR “diagnostic aid”[ti] OR "predictive rule*"[tiab] OR "predictive score*"[tiab] OR "prediction rule*"[tiab] OR "prediction score*"[tiab] OR “prediction model”[ti] OR "risk score*"[tiab]) NOT ("randomized" OR "nosocomial" OR "mechanical ventilation" OR "ventilator-associated" OR "COVID" OR “ARDS”[tiab] OR “gene”[ti] OR “stroke”[ti] OR “transplant*”[tiab])

Search adapted from: “Keogh C, Wallace E, O'Brien KK, et al. Developing an international register of clinical prediction rules for use in primary care: a descriptive analysis. Ann Fam Med 2014;12(4):360-366.

**Appendix B. Calibration plots**

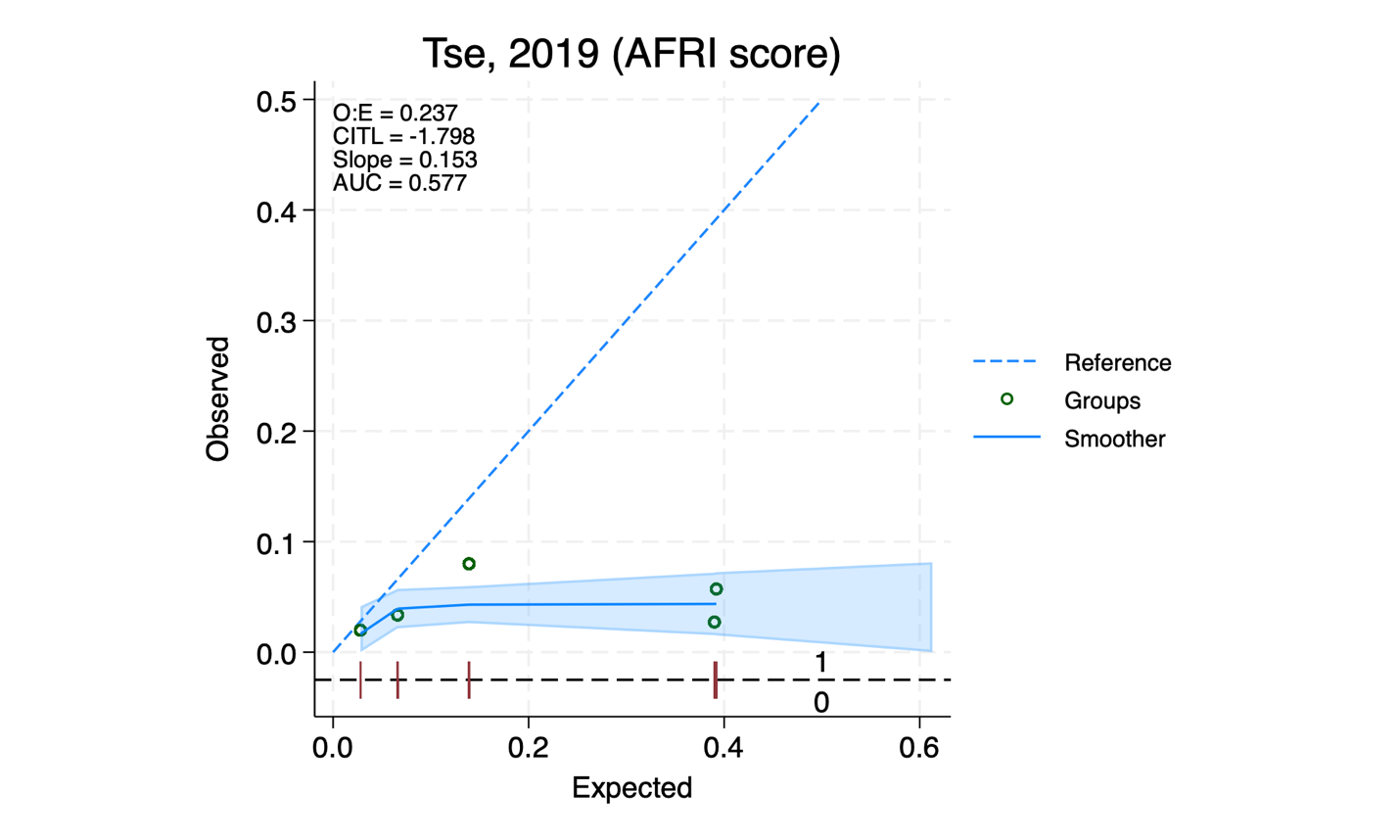


Appendix C.
